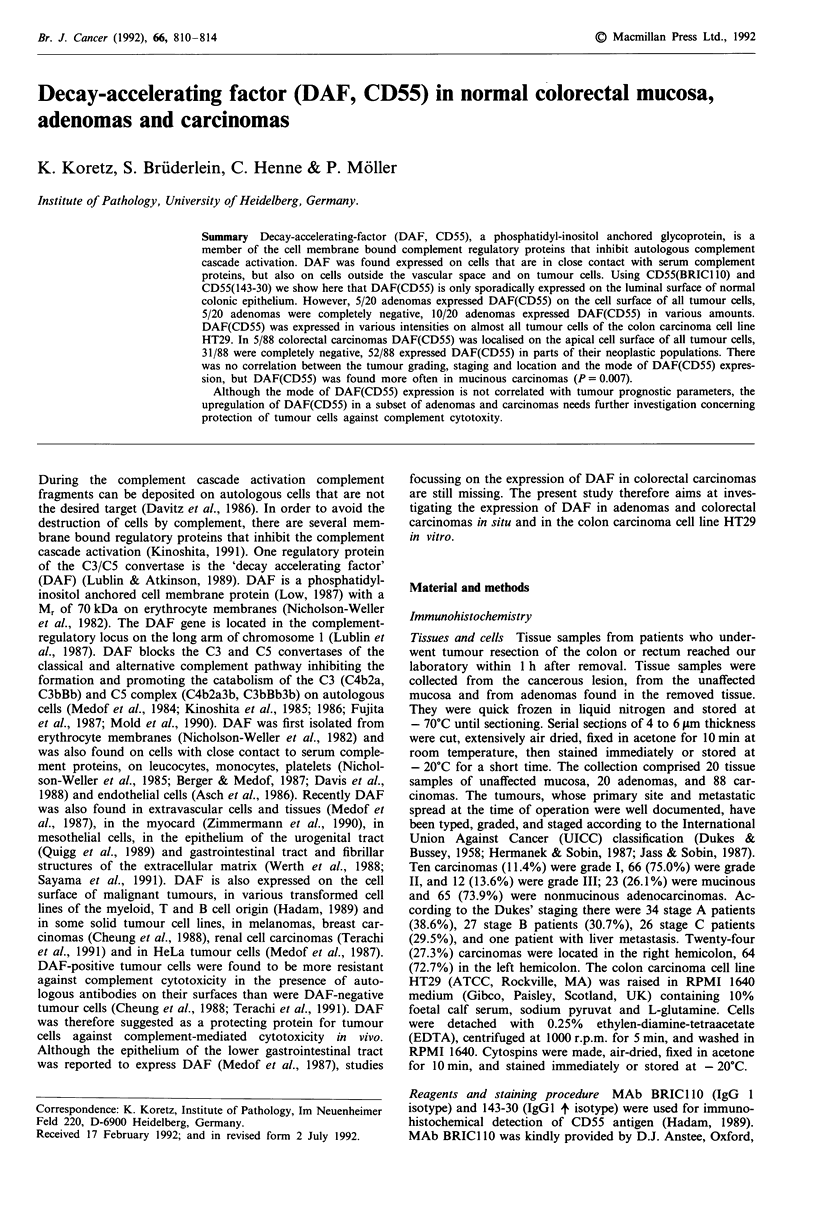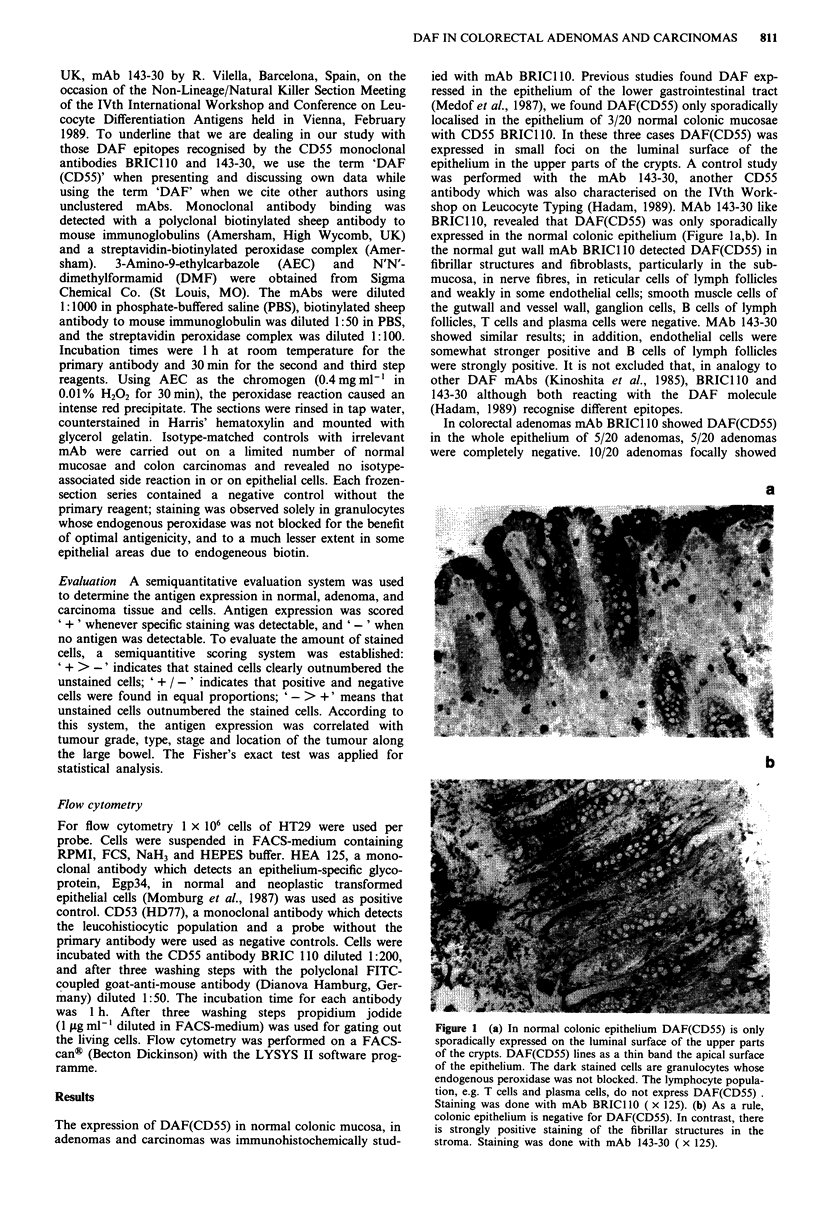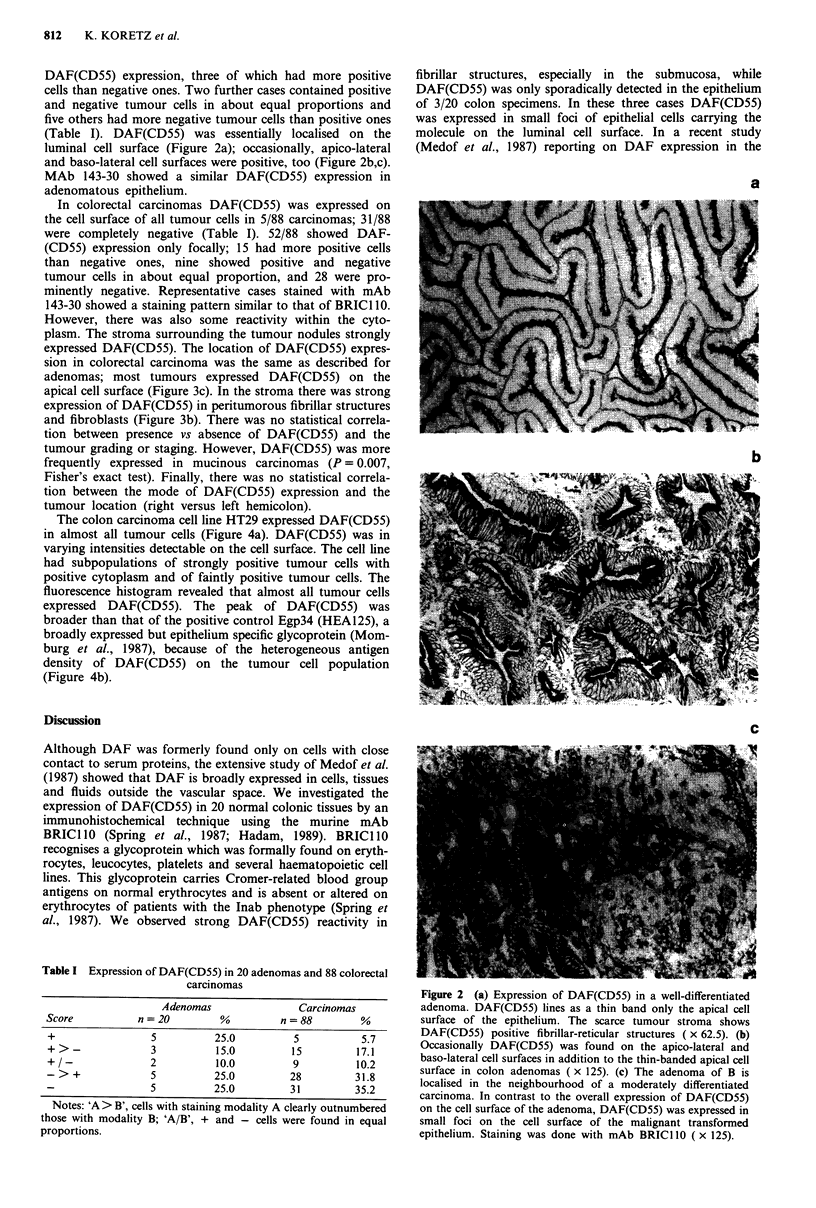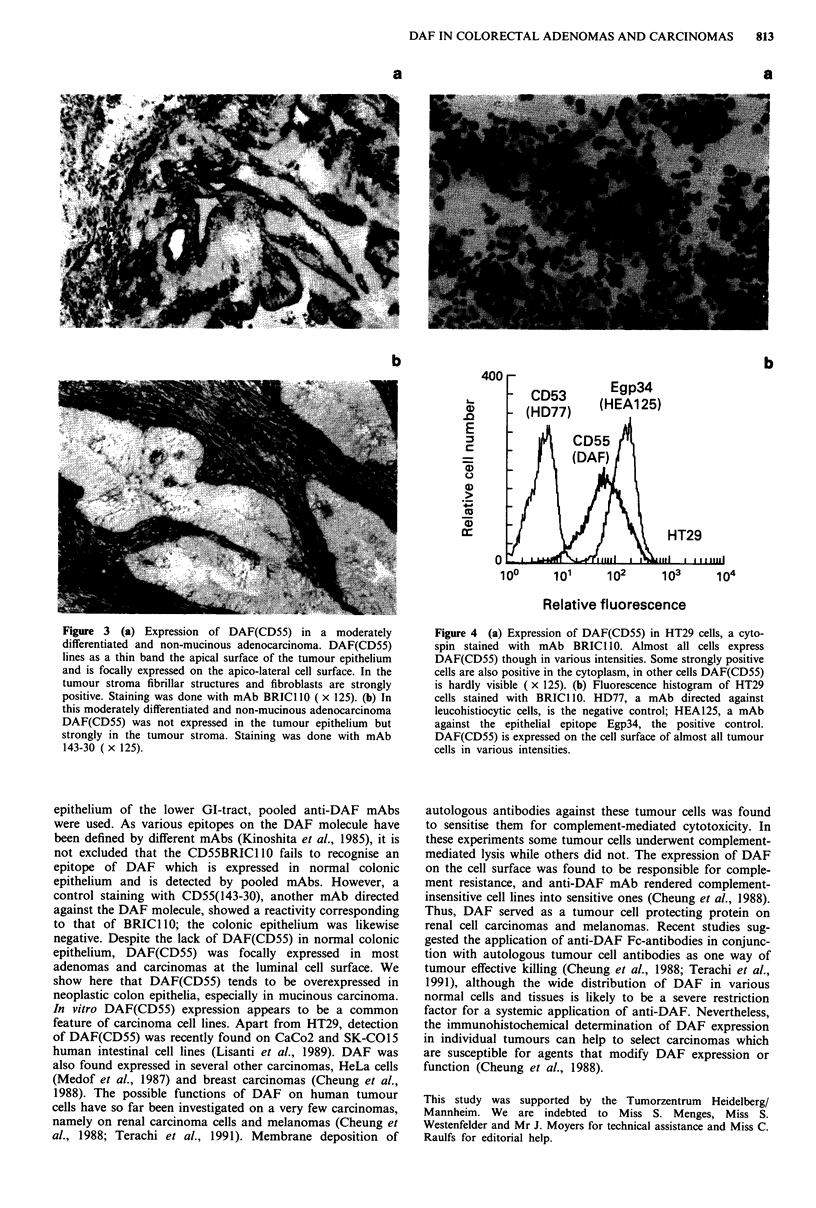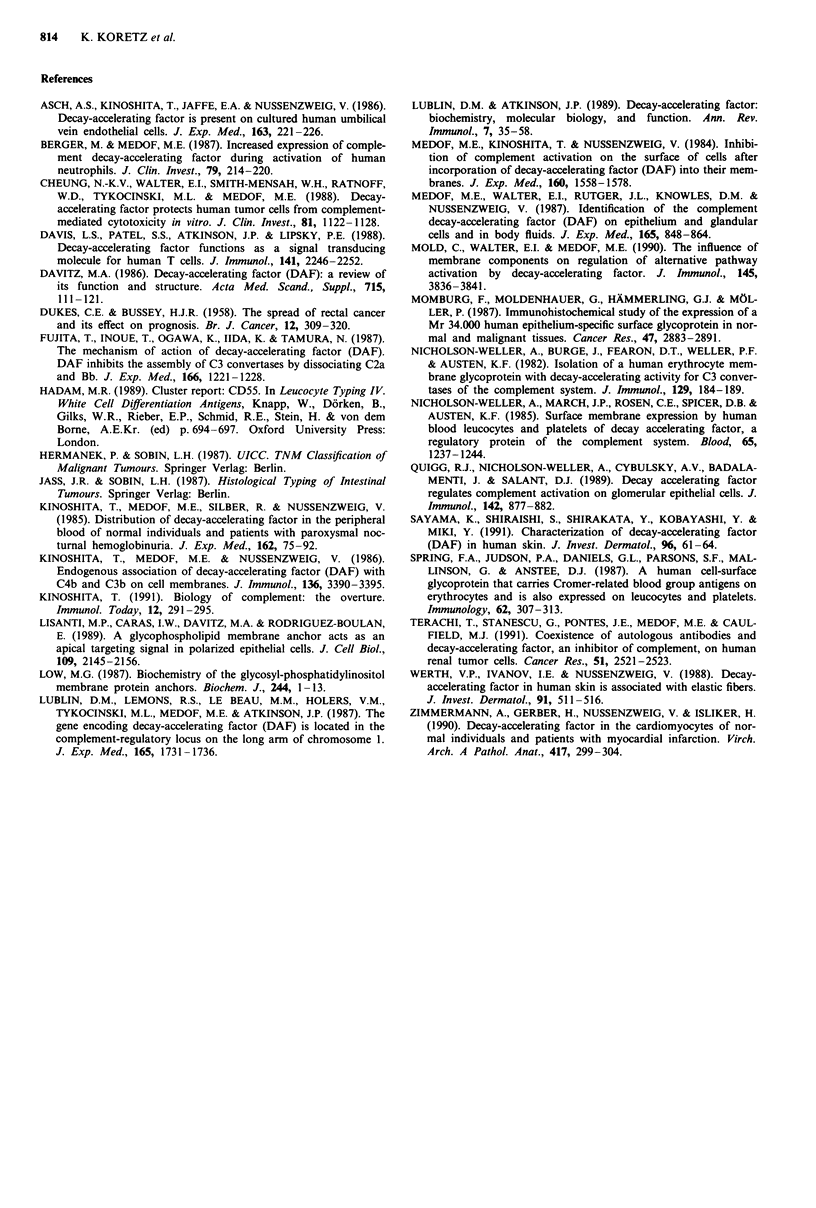# Decay-accelerating factor (DAF, CD55) in normal colorectal mucosa, adenomas and carcinomas.

**DOI:** 10.1038/bjc.1992.365

**Published:** 1992-11

**Authors:** K. Koretz, S. Brüderlein, C. Henne, P. Möller

**Affiliations:** Institute of Pathology, University of Heidelberg, Germany.

## Abstract

**Images:**


Br. J. Cancer (1992), 66, 810-814 © Macmillan Press Ltd., 1992

### Decay-accelerating factor (DAF, CD55) in normal colorectal mucosa, adenomas and carcinomas

K. Koretz, S. Brüderlein, C. Henne & P. Möller

*Institute of Pathology, University of Heidelberg, Germany.*

**Summary** Decay-accelerating-factor (DAF, CD55), a phosphatidyl-inositol anchored glycoprotein, is a member of the cell membrane bound complement regulatory proteins that inhibit autologous complement cascade activation. DAF was found expressed on cells that are in close contact with serum complement proteins, but also on cells outside the vascular space and on tumour cells. Using CD55(BRIC110) and CD55(143-30) we show here that DAF(CD55) is only sporadically expressed on the luminal surface of normal colonic epithelium. However, 5/20 adenomas expressed DAF(CD55) on the cell surface of all tumour cells, 5/20 adenomas were completely negative, 10/20 adenomas expressed DAF(CD55) in various amounts. DAF(CD55) was expressed in various intensities on almost all tumour cells of the colon carcinoma cell line HT29. In 5/88 colorectal carcinomas DAF(CD55) was localised on the apical cell surface of all tumour cells, 31/88 were completely negative, 52/88 expressed DAF(CD55) in parts of their neoplastic populations. There was no correlation between the tumour grading, staging and location and the mode of DAF(CD55) expression, but DAF(CD55) was found more often in mucinous carcinomas ($P = 0.007$).

Although the mode of DAF(CD55) expression is not correlated with tumour prognostic parameters, the upregulation of DAF(CD55) in a subset of adenomas and carcinomas needs further investigation concerning protection of tumour cells against complement cytotoxity.

During the complement cascade activation complement fragments can be deposited on autologous cells that are not the desired target (Davitz *et al.*, 1986). In order to avoid the destruction of cells by complement, there are several membrane bound regulatory proteins that inhibit the complement cascade activation (Kinoshita, 1991). One regulatory protein of the C3/C5 convertase is the 'decay accelerating factor' (DAF) (Lublin & Atkinson, 1989). DAF is a phosphatidyl-inositol anchored cell membrane protein (Low, 1987) with a $M_r$ of 70 kDa on erythrocyte membranes (Nicholson-Weller *et al.*, 1982). The DAF gene is located in the complement-regulatory locus on the long arm of chromosome 1 (Lublin *et al.*, 1987). DAF blocks the C3 and C5 convertases of the classical and alternative complement pathway inhibiting the formation and promoting the catabolism of the C3 (C4b2a, C3bBb) and C5 complex (C4b2a3b, C3bBb3b) on autologous cells (Medof *et al.*, 1984; Kinoshita *et al.*, 1985; 1986; Fujita *et al.*, 1987; Mold *et al.*, 1990). DAF was first isolated from erythrocyte membranes (Nicholson-Weller *et al.*, 1982) and was also found on cells with close contact to serum complement proteins, on leucocytes, monocytes, platelets (Nicholson-Weller *et al.*, 1985; Berger & Medof, 1987; Davis *et al.*, 1988) and endothelial cells (Asch *et al.*, 1986). Recently DAF was also found in extravascular cells and tissues (Medof *et al.*, 1987), in the myocard (Zimmermann *et al.*, 1990), in mesothelial cells, in the epithelium of the urogenital tract (Quigg *et al.*, 1989) and gastrointestinal tract and fibrillar structures of the extracellular matrix (Werth *et al.*, 1988; Sayama *et al.*, 1991). DAF is also expressed on the cell surface of malignant tumours, in various transformed cell lines of the myeloid, T and B cell origin (Hadam, 1989) and in some solid tumour cell lines, in melanomas, breast carcinomas (Cheung *et al.*, 1988), renal cell carcinomas (Terachi *et al.*, 1991) and in HeLa tumour cells (Medof *et al.*, 1987). DAF-positive tumour cells were found to be more resistant against complement cytotoxicity in the presence of autologous antibodies on their surfaces than were DAF-negative tumour cells (Cheung *et al.*, 1988; Terachi *et al.*, 1991). DAF was therefore suggested as a protecting protein for tumour cells against complement-mediated cytotoxicity *in vivo*. Although the epithelium of the lower gastrointestinal tract was reported to express DAF (Medof *et al.*, 1987), studies focussing on the expression of DAF in colorectal carcinomas are still missing. The present study therefore aims at investigating the expression of DAF in adenomas and colorectal carcinomas *in situ* and in the colon carcinoma cell line HT29 *in vitro*.

Correspondence: K. Koretz, Institute of Pathology, Im Neuenheimer Feld 220, D-6900 Heidelberg, Germany.
Received 17 February 1992; and in revised form 2 July 1992.

### Material and methods

### *Immunohistochemistry*

*Tissues and cells* Tissue samples from patients who underwent tumour resection of the colon or rectum reached our laboratory within 1 h after removal. Tissue samples were collected from the cancerous lesion, from the unaffected mucosa and from adenomas found in the removed tissue. They were quick frozen in liquid nitrogen and stored at − 70°C until sectioning. Serial sections of 4 to 6 μm thickness were cut, extensively air dried, fixed in acetone for 10 min at room temperature, then stained immediately or stored at − 20°C for a short time. The collection comprised 20 tissue samples of unaffected mucosa, 20 adenomas, and 88 carcinomas. The tumours, whose primary site and metastatic spread at the time of operation were well documented, have been typed, graded, and staged according to the International Union Against Cancer (UICC) classification (Dukes & Bussey, 1958; Hermanek & Sobin, 1987; Jass & Sobin, 1987). Ten carcinomas (11.4%) were grade I, 66 (75.0%) were grade II, and 12 (13.6%) were grade III; 23 (26.1%) were mucinous and 65 (73.9%) were nonmucinous adenocarcinomas. According to the Dukes' staging there were 34 stage A patients (38.6%), 27 stage B patients (30.7%), 26 stage C patients (29.5%), and one patient with liver metastasis. Twenty-four (27.3%) carcinomas were located in the right hemicolon, 64 (72.7%) in the left hemicolon. The colon carcinoma cell line HT29 (ATCC, Rockville, MA) was raised in RPMI 1640 medium (Gibco, Paisley, Scotland, UK) containing 10% foetal calf serum, sodium pyruvat and L-glutamine. Cells were detached with 0.25% ethylen-diamine-tetraacetate (EDTA), centrifuged at 1000 r.p.m. for 5 min, and washed in RPMI 1640. Cytospins were made, air-dried, fixed in acetone for 10 min, and stained immediately or stored at − 20°C.

*Reagents and staining procedure* MAb BRIC110 (IgG 1 isotype) and 143-30 (IgG1 ↑ isotype) were used for immunohistochemical detection of CD55 antigen (Hadam, 1989). MAb BRIC110 was kindly provided by D.J. Anstee, Oxford,

UK, mAb 143-30 by R. Vilella, Barcelona, Spain, on the occasion of the Non-Lineage/Natural Killer Section Meeting of the IVth International Workshop and Conference on Leucocyte Differentiation Antigens held in Vienna, February 1989. To underline that we are dealing in our study with those DAF epitopes recognised by the CD55 monoclonal antibodies BRIC110 and 143-30, we use the term 'DAF (CD55)' when presenting and discussing own data while using the term 'DAF' when we cite other authors using unclustered mAbs. Monoclonal antibody binding was detected with a polyclonal biotinylated sheep antibody to mouse immunoglobulins (Amersham, High Wycomb, UK) and a streptavidin-biotinylated peroxidase complex (Amersham). 3-Amino-9-ethylcarbazole (AEC) and N'N'-dimethylformamid (DMF) were obtained from Sigma Chemical Co. (St Louis, MO). The mAbs were diluted 1:1000 in phosphate-buffered saline (PBS), biotinylated sheep antibody to mouse immunoglobulin was diluted 1:50 in PBS, and the streptavidin peroxidase complex was diluted 1:100. Incubation times were 1 h at room temperature for the primary antibody and 30 min for the second and third step reagents. Using AEC as the chromogen (0.4 mg ml$^{-1}$ in 0.01% $H_2O_2$ for 30 min), the peroxidase reaction caused an intense red precipitate. The sections were rinsed in tap water, counterstained in Harris' hematoxylin and mounted with glycerol gelatin. Isotype-matched controls with irrelevant mAb were carried out on a limited number of normal mucosae and colon carcinomas and revealed no isotype-associated side reaction in or on epithelial cells. Each frozen-section series contained a negative control without the primary reagent; staining was observed solely in granulocytes whose endogenous peroxidase was not blocked for the benefit of optimal antigenicity, and to a much lesser extent in some epithelial areas due to endogeneous biotin.

*Evaluation* A semiquantitative evaluation system was used to determine the antigen expression in normal, adenoma, and carcinoma tissue and cells. Antigen expression was scored ' + ' whenever specific staining was detectable, and ' − ' when no antigen was detectable. To evaluate the amount of stained cells, a semiquantitive scoring system was established: ' + > − ' indicates that stained cells clearly outnumbered the unstained cells; ' + / − ' indicates that positive and negative cells were found in equal proportions; ' − > + ' means that unstained cells outnumbered the stained cells. According to this system, the antigen expression was correlated with tumour grade, type, stage and location of the tumour along the large bowel. The Fisher's exact test was applied for statistical analysis.

### *Flow cytometry*

For flow cytometry $1 \times 10^6$ cells of HT29 were used per probe. Cells were suspended in FACS-medium containing RPMI, FCS, $NaH_3$ and HEPES buffer. HEA 125, a monoclonal antibody which detects an epithelium-specific glycoprotein, Egp34, in normal and neoplastic transformed epithelial cells (Momburg *et al.*, 1987) was used as positive control. CD53 (HD77), a monoclonal antibody which detects the leucohistiocytic population and a probe without the primary antibody were used as negative controls. Cells were incubated with the CD55 antibody BRIC 110 diluted 1:200, and after three washing steps with the polyclonal FITC-coupled goat-anti-mouse antibody (Dianova Hamburg, Germany) diluted 1:50. The incubation time for each antibody was 1 h. After three washing steps propidium jodide (1 μg ml$^{-1}$ diluted in FACS-medium) was used for gating out the living cells. Flow cytometry was performed on a FACScan® (Becton Dickinson) with the LYSYS II software programme.

### Results

The expression of DAF(CD55) in normal colonic mucosa, in adenomas and carcinomas was immunohistochemically studied with mAb BRIC110. Previous studies found DAF expressed in the epithelium of the lower gastrointestinal tract (Medof *et al.*, 1987), we found DAF(CD55) only sporadically localised in the epithelium of 3/20 normal colonic mucosae with CD55 BRIC110. In these three cases DAF(CD55) was expressed in small foci on the luminal surface of the epithelium in the upper parts of the crypts. A control study was performed with the mAb 143-30, another CD55 antibody which was also characterised on the IVth Workshop on Leucocyte Typing (Hadam, 1989). MAb 143-30 like BRIC110, revealed that DAF(CD55) was only sporadically expressed in the normal colonic epithelium (Figure 1a,b). In the normal gut wall mAb BRIC110 detected DAF(CD55) in fibrillar structures and fibroblasts, particularly in the submucosa, in nerve fibres, in reticular cells of lymph follicles and weakly in some endothelial cells; smooth muscle cells of the gutwall and vessel wall, ganglion cells, B cells of lymph follicles, T cells and plasma cells were negative. MAb 143-30 showed similar results; in addition, endothelial cells were somewhat stronger positive and B cells of lymph follicles were strongly positive. It is not excluded that, in analogy to other DAF mAbs (Kinoshita *et al.*, 1985), BRIC110 and 143-30 although both reacting with the DAF molecule (Hadam, 1989) recognise different epitopes.

In colorectal adenomas mAb BRIC110 showed DAF(CD55) in the whole epithelium of 5/20 adenomas, 5/20 adenomas were completely negative. 10/20 adenomas focally showed

**Figure 1** (**a**) In normal colonic epithelium DAF(CD55) is only sporadically expressed on the luminal surface of the upper parts of the crypts. DAF(CD55) lines as a thin band the apical surface of the epithelium. The dark stained cells are granulocytes whose endogenous peroxidase was not blocked. The lymphocyte population, e.g. T cells and plasma cells, do not express DAF(CD55) . Staining was done with mAb BRIC110 ( × 125). (**b**) As a rule, colonic epithelium is negative for DAF(CD55). In contrast, there is strongly positive staining of the fibrillar structures in the stroma. Staining was done with mAb 143-30 ( × 125).

DAF(CD55) expression, three of which had more positive cells than negative ones. Two further cases contained positive and negative tumour cells in about equal proportions and five others had more negative tumour cells than positive ones (Table I). DAF(CD55) was essentially localised on the luminal cell surface (Figure 2a); occasionally, apico-lateral and baso-lateral cell surfaces were positive, too (Figure 2b,c). MAb 143-30 showed a similar DAF(CD55) expression in adenomatous epithelium.

In colorectal carcinomas DAF(CD55) was expressed on the cell surface of all tumour cells in 5/88 carcinomas; 31/88 were completely negative (Table I). 52/88 showed DAF-(CD55) expression only focally; 15 had more positive cells than negative ones, nine showed positive and negative tumour cells in about equal proportion, and 28 were prominently negative. Representative cases stained with mAb 143-30 showed a staining pattern similar to that of BRIC110. However, there was also some reactivity within the cytoplasm. The stroma surrounding the tumour nodules strongly expressed DAF(CD55). The location of DAF(CD55) expression in colorectal carcinoma was the same as described for adenomas; most tumours expressed DAF(CD55) on the apical cell surface (Figure 3c). In the stroma there was strong expression of DAF(CD55) in peritumorous fibrillar structures and fibroblasts (Figure 3b). There was no statistical correlation between presence *vs* absence of DAF(CD55) and the tumour grading or staging. However, DAF(CD55) was more frequently expressed in mucinous carcinomas ($P = 0.007$, Fisher's exact test). Finally, there was no statistical correlation between the mode of DAF(CD55) expression and the tumour location (right versus left hemicolon).

The colon carcinoma cell line HT29 expressed DAF(CD55) in almost all tumour cells (Figure 4a). DAF(CD55) was in varying intensities detectable on the cell surface. The cell line had subpopulations of strongly positive tumour cells with positive cytoplasm and of faintly positive tumour cells. The fluorescence histogram revealed that almost all tumour cells expressed DAF(CD55). The peak of DAF(CD55) was broader than that of the positive control Egp34 (HEA125), a broadly expressed but epithelium specific glycoprotein (Momburg *et al.*, 1987), because of the heterogeneous antigen density of DAF(CD55) on the tumour cell population (Figure 4b).

### Discussion

Although DAF was formerly found only on cells with close contact to serum proteins, the extensive study of Medof *et al.* (1987) showed that DAF is broadly expressed in cells, tissues and fluids outside the vascular space. We investigated the expression of DAF(CD55) in 20 normal colonic tissues by an immunohistochemical technique using the murine mAb BRIC110 (Spring *et al.*, 1987; Hadam, 1989). BRIC110 recognises a glycoprotein which was formally found on erythrocytes, leucocytes, platelets and several haematopoietic cell lines. This glycoprotein carries Cromer-related blood group antigens on normal erythrocytes and is absent or altered on erythrocytes of patients with the Inab phenotype (Spring *et al.*, 1987). We observed strong DAF(CD55) reactivity in fibrillar structures, especially in the submucosa, while DAF(CD55) was only sporadically detected in the epithelium of 3/20 colon specimens. In these three cases DAF(CD55) was expressed in small foci of epithelial cells carrying the molecule on the luminal cell surface. In a recent study (Medof *et al.*, 1987) reporting on DAF expression in the

**Table I** Expression of DAF(CD55) in 20 adenomas and 88 colorectal carcinomas

| | *Adenomas* | | *Carcinomas* | |
|---|---|---|---|---|
| *Score* | *n = 20* | *%* | *n = 88* | *%* |
| + | 5 | 25.0 | 5 | 5.7 |
| + > − | 3 | 15.0 | 15 | 17.1 |
| + / − | 2 | 10.0 | 9 | 10.2 |
| − > + | 5 | 25.0 | 28 | 31.8 |
| − | 5 | 25.0 | 31 | 35.2 |

Notes: 'A > B', cells with staining modality A clearly outnumbered those with modality B; 'A/B', + and − cells were found in equal proportions.

a b c

**Figure 2** (**a**) Expression of DAF(CD55) in a well-differentiated adenoma. DAF(CD55) lines as a thin band only the apical cell surface of the epithelium. The scarce tumour stroma shows DAF(CD55) positive fibrillar-reticular structures ( × 62.5). (**b**) Occasionally DAF(CD55) was found on the apico-lateral and baso-lateral cell surfaces in addition to the thin-banded apical cell surface in colon adenomas ( × 125). (**c**) The adenoma of B is localised in the neighbourhood of a moderately differentiated carcinoma. In contrast to the overall expression of DAF(CD55) on the cell surface of the adenoma, DAF(CD55) was expressed in small foci on the cell surface of the malignant transformed epithelium. Staining was done with mAb BRIC110 ( × 125).

a b

**Figure 3** (**a**) Expression of DAF(CD55) in a moderately differentiated and non-mucinous adenocarcinoma. DAF(CD55) lines as a thin band the apical surface of the tumour epithelium and is focally expressed on the apico-lateral cell surface. In the tumour stroma fibrillar structures and fibroblasts are strongly positive. Staining was done with mAb BRIC110 ( × 125). (**b**) In this moderately differentiated and non-mucinous adenocarcinoma DAF(CD55) was not expressed in the tumour epithelium but strongly in the tumour stroma. Staining was done with mAb 143-30 ( × 125).

a b

**Figure 4** (**a**) Expression of DAF(CD55) in HT29 cells, a cytospin stained with mAb BRIC110. Almost all cells express DAF(CD55) though in various intensities. Some strongly positive cells are also positive in the cytoplasm, in other cells DAF(CD55) is hardly visible ( × 125). (**b**) Fluorescence histogram of HT29 cells stained with BRIC110. HD77, a mAb directed against leucohistiocytic cells, is the negative control; HEA125, a mAb against the epithelial epitope Egp34, the positive control. DAF(CD55) is expressed on the cell surface of almost all tumour cells in various intensities.

epithelium of the lower GI-tract, pooled anti-DAF mAbs were used. As various epitopes on the DAF molecule have been defined by different mAbs (Kinoshita *et al.*, 1985), it is not excluded that the CD55BRIC110 fails to recognise an epitope of DAF which is expressed in normal colonic epithelium and is detected by pooled mAbs. However, a control staining with CD55(143-30), another mAb directed against the DAF molecule, showed a reactivity corresponding to that of BRIC110; the colonic epithelium was likewise negative. Despite the lack of DAF(CD55) in normal colonic epithelium, DAF(CD55) was focally expressed in most adenomas and carcinomas at the luminal cell surface. We show here that DAF(CD55) tends to be overexpressed in neoplastic colon epithelia, especially in mucinous carcinoma. *In vitro* DAF(CD55) expression appears to be a common feature of carcinoma cell lines. Apart from HT29, detection of DAF(CD55) was recently found on CaCo2 and SK-CO15 human intestinal cell lines (Lisanti *et al.*, 1989). DAF was also found expressed in several other carcinomas, HeLa cells (Medof *et al.*, 1987) and breast carcinomas (Cheung *et al.*, 1988). The possible functions of DAF on human tumour cells have so far been investigated on a very few carcinomas, namely on renal carcinoma cells and melanomas (Cheung *et al.*, 1988; Terachi *et al.*, 1991). Membrane deposition of autologous antibodies against these tumour cells was found to sensitise them for complement-mediated cytotoxicity. In these experiments some tumour cells underwent complement-mediated lysis while others did not. The expression of DAF on the cell surface was found to be responsible for complement resistance, and anti-DAF mAb rendered complement-insensitive cell lines into sensitive ones (Cheung *et al.*, 1988). Thus, DAF served as a tumour cell protecting protein on renal cell carcinomas and melanomas. Recent studies suggested the application of anti-DAF Fc-antibodies in conjunction with autologous tumour cell antibodies as one way of tumour effective killing (Cheung *et al.*, 1988; Terachi *et al.*, 1991), although the wide distribution of DAF in various normal cells and tissues is likely to be a severe restriction factor for a systemic application of anti-DAF. Nevertheless, the immunohistochemical determination of DAF expression in individual tumours can help to select carcinomas which are susceptible for agents that modify DAF expression or function (Cheung *et al.*, 1988).

This study was supported by the Tumorzentrum Heidelberg/Mannheim. We are indebted to Miss S. Menges, Miss S. Westenfelder and Mr J. Moyers for technical assistance and Miss C. Raulfs for editorial help.

### References

ASCH, A.S., KINOSHITA, T., JAFFE, E.A. & NUSSENZWEIG, V. (1986). Decay-accelerating factor is present on cultured human umbilical vein endothelial cells. *J. Exp. Med.*, **163**, 221–226.

BERGER, M. & MEDOF, M.E. (1987). Increased expression of complement decay-accelerating factor during activation of human neutrophils. *J. Clin. Invest.*, **79**, 214–220.

CHEUNG, N.-K.V., WALTER, E.I., SMITH-MENSAH, W.H., RATNOFF, W.D., TYKOCINSKI, M.L. & MEDOF, M.E. (1988). Decay-accelerating factor protects human tumor cells from complement-mediated cytotoxicity *in vitro*. *J. Clin. Invest.*, **81**, 1122–1128.

DAVIS, L.S., PATEL, S.S., ATKINSON, J.P. & LIPSKY, P.E. (1988). Decay-accelerating factor functions as a signal transducing molecule for human T cells. *J. Immunol.*, **141**, 2246–2252.

DAVITZ, M.A. (1986). Decay-accelerating factor (DAF): a review of its function and structure. *Acta Med. Scand., Suppl.*, **715**, 111–121.

DUKES, C.E. & BUSSEY, H.J.R. (1958). The spread of rectal cancer and its effect on prognosis. *Br. J. Cancer*, **12**, 309–320.

FUJITA, T., INOUE, T., OGAWA, K., IIDA, K. & TAMURA, N. (1987). The mechanism of action of decay-accelerating factor (DAF). DAF inhibits the assembly of C3 convertases by dissociating C2a and Bb. *J. Exp. Med.*, **166**, 1221–1228.

HADAM, M.R. (1989). Cluster report: CD55. In *Leucocyte Typing IV. White Cell Differentiation Antigens*, Knapp, W., Dörken, B., Gilks, W.R., Rieber, E.P., Schmid, R.E., Stein, H. & von dem Borne, A.E.Kr. (ed) p. 694–697. Oxford University Press: London.

HERMANEK, P. & SOBIN, L.H. (1987). *UICC. TNM Classification of Malignant Tumours*. Springer Verlag: Berlin.

JASS, J.R. & SOBIN, L.H. (1987). *Histological Typing of Intestinal Tumours*. Springer Verlag: Berlin.

KINOSHITA, T., MEDOF, M.E., SILBER, R. & NUSSENZWEIG, V. (1985). Distribution of decay-accelerating factor in the peripheral blood of normal individuals and patients with paroxysmal nocturnal hemoglobinuria. *J. Exp. Med.*, **162**, 75–92.

KINOSHITA, T., MEDOF, M.E. & NUSSENZWEIG, V. (1986). Endogenous association of decay-accelerating factor (DAF) with C4b and C3b on cell membranes. *J. Immunol.*, **136**, 3390–3395.

KINOSHITA, T. (1991). Biology of complement: the overture. *Immunol. Today*, **12**, 291–295.

LISANTI, M.P., CARAS, I.W., DAVITZ, M.A. & RODRIGUEZ-BOULAN, E. (1989). A glycophospholipid membrane anchor acts as an apical targeting signal in polarized epithelial cells. *J. Cell Biol.*, **109**, 2145–2156.

LOW, M.G. (1987). Biochemistry of the glycosyl-phosphatidylinositol membrane protein anchors. *Biochem. J.*, **244**, 1–13.

LUBLIN, D.M., LEMONS, R.S., LE BEAU, M.M., HOLERS, V.M., TYKOCINSKI, M.L., MEDOF, M.E. & ATKINSON, J.P. (1987). The gene encoding decay-accelerating factor (DAF) is located in the complement-regulatory locus on the long arm of chromosome 1. *J. Exp. Med.*, **165**, 1731–1736.

LUBLIN, D.M. & ATKINSON, J.P. (1989). Decay-accelerating factor: biochemistry, molecular biology, and function. *Ann. Rev. Immunol.*, **7**, 35–58.

MEDOF, M.E., KINOSHITA, T. & NUSSENZWEIG, V. (1984). Inhibition of complement activation on the surface of cells after incorporation of decay-accelerating factor (DAF) into their membranes. *J. Exp. Med.*, **160**, 1558–1578.

MEDOF, M.E., WALTER, E.I., RUTGER, J.L., KNOWLES, D.M. & NUSSENZWEIG, V. (1987). Identification of the complement decay-accelerating factor (DAF) on epithelium and glandular cells and in body fluids. *J. Exp. Med.*, **165**, 848–864.

MOLD, C., WALTER, E.I. & MEDOF, M.E. (1990). The influence of membrane components on regulation of alternative pathway activation by decay-accelerating factor. *J. Immunol.*, **145**, 3836–3841.

MOMBURG, F., MOLDENHAUER, G., HÄMMERLING, G.J. & MÖLLER, P. (1987). Immunohistochemical study of the expression of a Mr 34.000 human epithelium-specific surface glycoprotein in normal and malignant tissues. *Cancer Res.*, **47**, 2883–2891.

NICHOLSON-WELLER, A., BURGE, J., FEARON, D.T., WELLER, P.F. & AUSTEN, K.F. (1982). Isolation of a human erythrocyte membrane glycoprotein with decay-accelerating activity for C3 convertases of the complement system. *J. Immunol.*, **129**, 184–189.

NICHOLSON-WELLER, A., MARCH, J.P., ROSEN, C.E., SPICER, D.B. & AUSTEN, K.F. (1985). Surface membrane expression by human blood leucocytes and platelets of decay accelerating factor, a regulatory protein of the complement system. *Blood*, **65**, 1237–1244.

QUIGG, R.J., NICHOLSON-WELLER, A., CYBULSKY, A.V., BADALAMENTI, J. & SALANT, D.J. (1989). Decay accelerating factor regulates complement activation on glomerular epithelial cells. *J. Immunol.*, **142**, 877–882.

SAYAMA, K., SHIRAISHI, S., SHIRAKATA, Y., KOBAYASHI, Y. & MIKI, Y. (1991). Characterization of decay-accelerating factor (DAF) in human skin. *J. Invest. Dermatol.*, **96**, 61–64.

SPRING, F.A., JUDSON, P.A., DANIELS, G.L., PARSONS, S.F., MALLINSON, G. & ANSTEE, D.J. (1987). A human cell-surface glycoprotein that carries Cromer-related blood group antigens on erythrocytes and is also expressed on leucocytes and platelets. *Immunology*, **62**, 307–313.

TERACHI, T., STANESCU, G., PONTES, J.E., MEDOF, M.E. & CAULFIELD, M.J. (1991). Coexistence of autologous antibodies and decay-accelerating factor, an inhibitor of complement, on human renal tumor cells. *Cancer Res.*, **51**, 2521–2523.

WERTH, V.P., IVANOV, I.E. & NUSSENZWEIG, V. (1988). Decay-accelerating factor in human skin is associated with elastic fibers. *J. Invest. Dermatol.*, **91**, 511–516.

ZIMMERMANN, A., GERBER, H., NUSSENZWEIG, V. & ISLIKER, H. (1990). Decay-accelerating factor in the cardiomyocytes of normal individuals and patients with myocardial infarction. *Virch. Arch. A Pathol. Anat.*, **417**, 299–304.